# The Evolution of Pepsinogen C Genes in Vertebrates: Duplication, Loss and Functional Diversification

**DOI:** 10.1371/journal.pone.0032852

**Published:** 2012-03-09

**Authors:** Luís Filipe Costa Castro, Monica Lopes-Marques, Odete Gonçalves, Jonathan Mark Wilson

**Affiliations:** 1 CIMAR Associate Laboratory, CIIMAR–Interdisciplinary Centre of Marine and Environmental Research, UPorto–University of Porto, Porto, Portugal; 2 Institute of Biomedical Sciences Abel Salazar (ICBAS), University of Porto, Porto, Portugal; University of Wyoming, United States of America

## Abstract

**Background:**

Aspartic proteases comprise a large group of enzymes involved in peptide proteolysis. This collection includes prominent enzymes globally categorized as pepsins, which are derived from pepsinogen precursors. Pepsins are involved in gastric digestion, a hallmark of vertebrate physiology. An important member among the pepsinogens is pepsinogen C (*Pgc*). A particular aspect of *Pgc* is its apparent single copy status, which contrasts with the numerous gene copies found for example in pepsinogen A (*Pga*). Although gene sequences with similarity to *Pgc* have been described in some vertebrate groups, no exhaustive evolutionary framework has been considered so far.

**Methodology/Principal Findings:**

By combining phylogenetics and genomic analysis, we find an unexpected *Pgc* diversity in the vertebrate sub-phylum. We were able to reconstruct gene duplication timings relative to the divergence of major vertebrate clades. Before tetrapod divergence, a single *Pgc* gene tandemly expanded to produce two gene lineages (*Pgbc* and *Pgc2*). These have been differentially retained in various classes. Accordingly, we find *Pgc2* in sauropsids, amphibians and marsupials, but not in eutherian mammals. *Pgbc* was retained in amphibians, but duplicated in the ancestor of amniotes giving rise to *Pgb* and *Pgc1*. The latter was retained in mammals and probably in reptiles and marsupials but not in birds. *Pgb* was kept in all of the amniote clade with independent episodes of loss in some mammalian species. Lineage specific expansions of *Pgc2* and *Pgbc* have also occurred in marsupials and amphibians respectively. We find that teleost and tetrapod *Pgc* genes reside in distinct genomic regions hinting at a possible translocation.

**Conclusions:**

We conclude that the repertoire of *Pgc* genes is larger than previously reported, and that tandem duplications have modelled the history of *Pgc* genes. We hypothesize that gene expansion lead to functional divergence in tetrapods, coincident with the invasion of terrestrial habitats.

## Introduction

Pepsinogens, the precursors of pepsins, are a group of aspartic proteases involved in the specific hydrolysis of peptides. Typically, they show a high and localized expression in the stomach due to their crucial role in protein digestion. After secretion into the gastric lumen, the pepsinogens are activated into pepsins by the action of hydrochloric acid, which alters their structural conformation [1]. The activation involves the autocatalytic cleavage of the prosegment from the N-terminus of the enzyme [1].

A wide diversity of pepsinogen genes is found in mammalian species. The five group nomenclature distinguishes pepsinogen A (*Pga*), B (*Pgb*), C (*Pgc*), F (*Pgf*), and prochymosin (*Cym*) [2], [3]. The various pepsinogen gene families are thought to have emerged from a common intracellular aspartic protease through gene duplication, though the exact duplication timings and processes are presently unknown [4], [5]. Within each pepsinogen family, gene numbers vary significantly between species and gene family. For example, *Pga* has three gene copies in humans, while a single copy is found in the opossum [6]. An extreme case of lineage-specific gene expansion was recently determined in the orangutan where fourteen different *Pga* cDNAs were found, corresponding to a minimum of eight *loci* [7]. This is in sharp contrast to the condition observed in the *Pgc* gene family, which is mostly considered single copy [3]. Therefore, *Pgc* has been suggested as a reliable molecular marker in species phylogenetic analysis [2], [8]. This distinctive feature of *Pgc* is apparently corroborated by the characterization of single cDNAs in vertebrate classes such as teleosts [9] amphibians [10] and birds [11]. Contradictorily, Ordoñez et al. [6] have suggested the presence of extra *Pgc-like* sequences in some vertebrate species. Nevertheless, no phylogenetics or comprehensive species sampling was performed thus preventing elaboration on duplication timings/processes or evolutionary history. For example, it was argued that *Pgb* and *Pgc* derived from tandem gene duplication in the therian mammalian ancestor [6], a proposal impossible to confirm without phylogeny. In this study, we set out to investigate the Pgc, a gene family which together with other pepsinogen isoforms is fundamental for the vertebrate gastric function. We take an approach that combines phylogenetics and comparative genomics to unravel a complex evolutionary pathway of *Pgc* in the vertebrate sub-phylum. We find that contrary to previous reports, the diversity of the *Pgc* gene family is broader with various episodes of gene duplication and loss, particularly in tetrapods. Based on the current findings we recommend a new gene nomenclature for *Pgc* genes which incorporates gene duplication history and phylogenetic distribution.

## Methods

### Identification of *Pgc* genes

**Figure 1:**
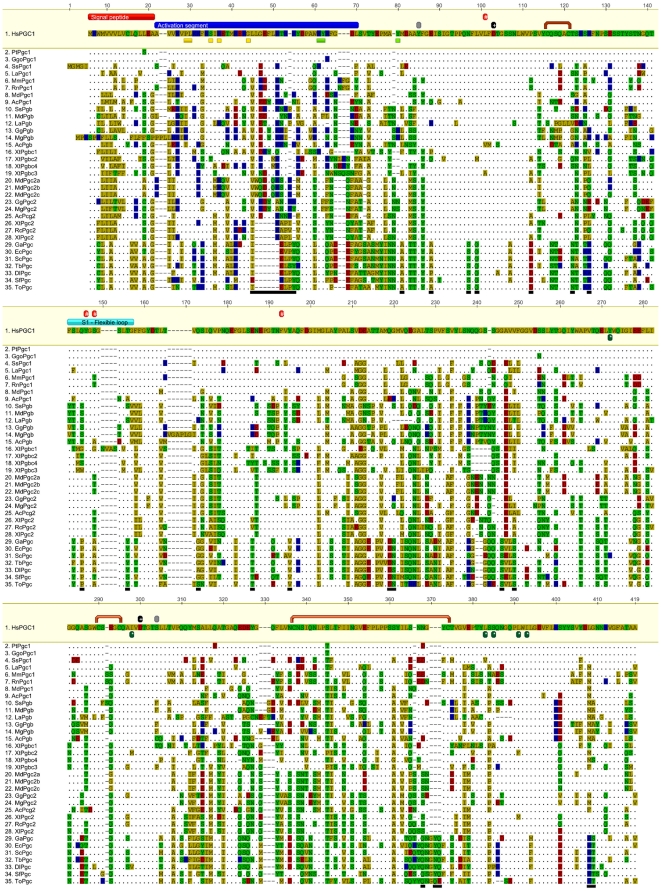
Multiple sequence alignment of vertebrate *Pgc-like* sequences performed in Geneious V5.5.6 using Clustal plugin with Gonnet scoring matrix and the following parameters: Gap opening = 10, Gap extension = 0.2. The red bar indicates signal peptide, blue bar activation segment or propeptide, yellow boxes highly conserved residues of the propeptide [3], light green boxes residues (pLys37 pTyr38 and Tyr9) involved in interactions that block access to the catalytic aspartates at neutral pH [1] black boxes “+” conserved catalytic aspartates (Asp32 and Asp217), orange bridges six conserved cysteines involved in the formation of disulphide linkages (Cys45, Cys50, Cys208, Cys212, Cys251, Cys284), grey boxes residues reported to be involved in pepsinogen B substrate specifity [13] and underlining black boxes sequence features specific to fish pepsinogens (All coordinates are relative to human PGC).

*Pgc* sequences were identified in the Ensembl and GenBank databases for the following species with genome sequences available: *Homo sapiens* (human), *Pan troglodytes* (common chimpanzee), *Gorilla gorilla* (Gorilla), *Loxodonta fricana* (African savanna elephant), *Sus scrofa* (pig), *Mus musculus* (mouse), *Rattus norvegicus* (brown rat), *Monodelphis domestica* (opossum), *Xenopus tropicalis* (western clawed frog), *Anolis carolinensis* (anolis), *Gallus gallus* (chicken), *Meleagris gallopavo* (turkey), *Tetraodon nigroviridis* (green spotted puffer), *Takifugu rubripes* (pufferfish), *Danio rerio* (zebrafish), *Oryzias latipes* (medaka) and *Gasterosteus aculeatus* (stickleback). To identify non-annotated genes Blastp searches were performed using the human PGC protein sequence. Blast searches to EST databases (when available) were also implemented. Sequences previously described in organisms (teleosts) without genome sequences were also incorporated in the phylogenetic analysis. Accession numbers for the sequences are listed in Table 1. The alignment provided in Fig. 1 was performed in Geneious V5.4.6 [12] with the Clustal plugin (settings below).

**Table 1:** List of accession numbers for all the *Pgc* sequences used in the phylogenetic analysis.

Species	Accession number	Name	Pro-segment PI	Moietyc PI
*Homo sapiens*	NP_002621	*PGC1*	10.67	3.35
*Pan troglodytes*	XP_518465	*Pgc1*	10.67	3.31
*Gorilla gorilla*	ENSGGOP00000002651	*Pgc1*	10.48	3.35
*Loxodonta africana*	ENSLAFP00000025164	*Pgb*	10.11	4.16
	ENSLAFP00000014376	*Pgc1*	10.60	3.54
*Sus scrofa*	XP_003355296	*Pgb*	10.28	3.62
	XP_003128442	*Pgc1*	*10.67*	3.51
*Mus musculus*	NP_080249	*Pgc1*	*10.47*	3.50
*Rattus norvegicus*	NP_579818	*Pgc1*	*10.40*	3.46
*Monodelphis domestica*	NP_001028152	*Pgb*	*10.28*	3.68
	XP_001370482	*Pgc2c*	*10.92*	3.26
	XP_001370462	*Pgc2b*	*10.92*	3.38
	XP_001370404	*Pgc1*	*10.14*	3.49
	XP_001370435	*Pgc2a*	*10.92*	3.38
*Meleagris gallopavo*	ENSMGAP00000018167	*Pgb*	*10.91*	4.83
	ENSMGAP00000006238	*Pgc2*	*10.15*	3.23
*Gallus gallus*	XP_425832	*Pgb*	*11.00*	4.24
	NP_990208	*Pgc2*	*10.29*	3.14
*Anolis carolinensis*	XP_003220378.1	*Pgb*	*10.06*	3.61
	Gene ID:100567329	*ΨPgb*	n.a	n.a
	XP_003220379.1	*Pgc1*	*9.61*	3.92
	XP_003220377.1	*Pgc2*	*10.35*	3.09
*Xenopus tropicalis*	XM_002932980	*Pgc2*	*10.54*	3.21
	XM_002932982	*Pgbc1*	*10.64*	3.68
	NM_001030432	*Pgbc2*	*10.84*	3.56
	NM_001015682	*Pgbc3*	*11.08*	3.52
	NM_001032309	*Pgbc4*	*10.78*	3.50
*Xenopus laevis*	AB045379	*Pgc2*	*10.54*	3.26
*Rana catesbeiana*	M73750	*Pgc2*	*10.63*	3.28
*Gasterosteus aculeatus*	ENSGACG00000012388	*Pgc3*	*9.06*	3.85
*Epinephelus coioides*	EU136029	*Pgc3*	*8.99*	4.17
*Siniperca chuatsi*	FJ463157	*Pgc3*	*9.77*	3.74
*Trematomus bernacchii*	AJ550952	*Pgc3*	*9.08*	4.53
*Dicentrarchus labrax*	EF690286	*Pgc3*	*9.63*	4.08
*Salvelinus fontinalis*	AF275939	*Pgc3*	*9.09*	3.69
*Thunnus orientalis*	AB440202	*Pgc3*	*9.86*	4.08

Also shown is the isolelectric point (PI) of the pro-segment and of the pepsin moiety.

### Phylogenetic analysis

PGC amino acid sequences were aligned in ClustalX 2.0.11 with standard settings (Gonnet weight matrix, gap opening = 10 and gap extension = 0.2) [13], [14]. All positions containing gaps and missing data were eliminated. The final dataset involved 42 amino acid sequences and 339 positions. The evolutionary history was inferred using two methods. A Neighbor-Joining (NJ) tree [15] was reconstructed using standard settings with ClustalX 2.0.11 [13], [14]. The robustness of the tree was assessed through 1000 bootstrap replicates of the data. The same alignment was also used to generate a Maximum likelihood tree (ML). The evolutionary model was derived from ProtTest (LG+I+G+F) [16]. The ML tree was reconstructed using PhyML online [17] with the amino acid frequency (equilibrium frequency), proportion of invariable sites and gamma-shape (4 rate substitution categories) for the amino acid substitution rate heterogeneity parameters estimated from the dataset. Bootstrap analysis (1000 replicates) was carried out to determine the robustness of the tree. The pufferfish *Pga* sequence was used as an outgroup in TreeView 1.6.6.

### Comparative genomics and neighboring gene families

The chromosomal location of the *Pgc* genes and the flanking gene families was collected from the Ensembl and GenBank databases. The human *PGC* and *PGBψloci* were used as the tetrapod genomic models for comparison with the stickleback. Information on the evolutionary history (orthologous *versus* paralogous) of the gene families flanking human and stickleback *Pgc loci* was collected from the Ensembl paralogue and orthologue prediction pipeline.

### Structural comparative modeling

The crystal structure of the *H. sapiens* progastricsin (1HTR) [18] was used as a template for 3D modeling. To predict structure a Modeller algorithm [19] available at HHpred was used [20], [21] The predicted structural models were evaluated using modeller output Verify 3D [22] and in all cases accurate structures were achieved. Structural visualization and analysis was performed using Open-Source PyMOL V1.3. (academic version) [23].

## Results

### An unexpected diversity of *Pgc* genes in tetrapods

We initiated this research by first establishing whether the single copy status of *Pgc* is a typical feature of this gene family. All *Pgc* sequences retrieved from database search are listed in Table 1. In the investigated mammalian species we find three dissimilar gene complements. Human, gorilla, chimpanzee, mouse and rat all have a single *Pgc* sequence, while the African elephant and pig have two. In humans a second *Pgc-like* sequence, named *Pgb*, is also found though this is a pseudogene [6]. In contrast, the opossum has five identifiable open reading frames (ORFs) with similarity to the *Pgc* gene family. Birds represented here by the chicken and turkey, have two *Pgc-like* sequences, whereas in the lizard we have uncovered three. A fourth sequence is present in the reptile genome but the current assembly indicates a frameshift mutation in the eighth exon producing a truncated protein (Figure S1). Whether this is the first step of pseudogenization or a sequencing error remains to be determined. The western clawed frog has the same number of *Pgc-like* sequences to that found in the opossum. In teleosts we find two distinct situations. Whilst in the stickleback a single representative of *Pgc* was recovered, other fish species with available genome sequences have no identifiable hits to *Pgc*.

We next performed sequence alignment of the all the collected sequences (Fig. 1). In all sequences three distinct regions can be distinguished: the signal peptide (1–16 human; PGC coordinates used hereafter, Fig. 1 red bar); the activation peptide (residues 16–59, Fig. 1 blue bar), and the active enzyme moiety (residues 59–388). In the activation segment (prosegment) highly conserved residues were observed (Fig. 1 yellow boxes 1). These residues (pLeu7, pSer12, pArg14, pGly21 and pLys37 - p prosegment numbering) are also conserved in PGA and PGY suggesting that they play an important role in the activation segment [3], [24]. In fact pepsinogens are activated by the cleavage of the prosegment. There are two major cleavage sites in human PGC, one located between pPhe26 and pLeu27 and the second located at the last residue of the prosegment pLeu43 and the first residue of the enzyme moiety Ser1 [1], [3], [25]. In a neutral pH the prosegment is coupled to the enzyme moiety by electrostatic interactions and hydrogen-bonds, pLys37, pTyr38 and Tyr9 (Fig. 1 green boxes) bind to the catalytic aspartates (Fig. 1 black boxes “+”) [1], [26], [27], [28] In an acidic pH environment acidic residues in the enzyme moiety become protonated disrupting electrostatic interactions with the prosegment (which has a basic character), releasing the prosegment for proteolytic cleavage and enzyme activation [3], [29], [30]. In fish pepsinogens a deletion of several residues in the prosegment is observed (Fig. 1 activation segment, lower black bar) leads to a decrease in the number of basic residues in the prosegment, and given the PI values for each enzyme region (Table 1), we deduced that the activation of fish pepsinogens occurs in conditions that are comparatively more alkaline. In accordance, the analysis of the PI of each enzyme moiety supports this observation (Table 1). While tetrapod *Pgc* have values below 3,5 (with the exception of *Pgb*), teleost *Pgc* are mostly above 3,6 and closer to 4, suggesting distinct activation conditions. Furthermore, the cleavage of the prosegment can be completed in the sequential pathway or in a direct pathway [1]. In human the sequential pathway involves an initial cleavage between pPhe26-pLeu27 bond followed by the cleavage of the prosegment from the active enzyme between residues pLeu43 and Ser1 (Fig. 1 Green boxes). Given that pLeu27 is deleted in fish and western clawed frog prosegment and pPhe26 is not conserved in fish, lizard, birds and western clawed frog, a direct or distinct activation pathway is expected for these species [1], [3], [25].

All PGC-like sequences analysed here present the highly conserved catalytic-site aspartates, Asp32 and Asp217, characteristic of the aspartic protease family (Fig. 1, black boxes “+”) and the six conserved cysteines reported to be involved in the formation of three bisulfide bridges (Fig. 1 orange bridges, Cys45–Cys50; Cys208–Cys212 and Cys251–Cys284). Although the elephant PGB presents a Serine at position 45 corresponding to the first Cysteine, this bridge (Cys45–Cys50) has been reported as unessential in the correct protein folding [31].

The catalytic-site aspartates are found in a substrate binding cleft in the enzyme moiety, and bordered by S1 and S1′ subsites (Fig. 1 red boxes “#” and dark green boxes “*”, respectively). These subsites are involved in the binding of the substrate to the enzyme and have been reported to play an essential role in substrate specificity [32]. The S1 subsite is highly conserved in all PGC-like sequences and is located near the Asp32, presenting a flexible loop (Fig. 1, Light blue box “S1- Flexible loop”) formed by several residues namely Phe71-Gly81, Leu30 Tyr75, Ser77 and Phe112 [1], [3].

The S1′subsite, which is located in the neighbourhood of Asp217 and is formed by the following residues Tyr190, Ile215, Leu293, Ser295, Leu301 and Ile303, (Fig. 1 dark green boxes “*”) and is also highly conserved between the analysed sequences, although neighbouring residues may vary. The S1 and S1′ subsites show distinct degrees of conservation. In fact the S1 subsite is comparatively less conserved. This may be related to the fact that the S1 subsite has been reported to play an important role in substrate binding [32]. Therefore, a higher residue variation at this site suggests diversification in the substrate cleft in order to accommodate distinct substrates.

Considering the S1′ subsite in detail we can observe residue patterns that are characteristic of a determined pepsinogen group. For example all PGB-like sequences present a valine residue at position 82 (Fig. 1 light blue box “S1 Flexible loop”) along with this amino acid, PGB-like sequences tend to present a threonine at position 72 and a serine at position 74. Concerning the fish PGC (PGC), it is possible to observe that in the S1 subsite there are several distinctive features. All fish PGC sequences present an aromatic residue tyrosine at position 72 (phenylalanine in the case of Tb), which in other species is generally a serine or a threonine. In addition, at position 81 fish PGC have a tyrosine or serine in contrast to a highly conserved threonine in all other sequences and an exclusive proline is found at position 74. This subtle distinct residue composition in the S1 subsite of the PGC-like sequences may lead to distinct network of hydrogen bonds likely shifting substrate specificities.

### Phylogenetic analysis indicates multiple events of *Pgc* gene duplication

**Figure 2:**
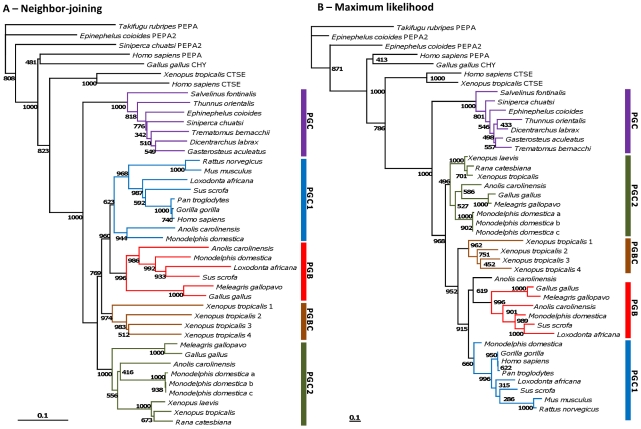
Neighbor-Joining (A) and Maximum likelihood (B) tree of the *Pgc* gene family. Values at nodes are bootstrap values (1000).

The finding of numerous *Pgc-like* sequences per species is surprising given previous reports arguing its single copy condition [3]. To clarify the evolutionary relationships between the various sequences as well as the duplication timings, we next constructed phylogenetic trees with NJ and ML (Fig. 2A and Fig. 2B). Both tree reconstructions show similar relationships between the retrieved sequences, with some differences. We find that classical *Pgc* (hereafter renamed *Pgc1*) and *Pgb* genes are found not only in the mammalian lineage as previously suggested [6]. A strongly supported *Pgb* clade includes a sequence also from birds and the reptile with both phylogenetic methods. The phylogenetic placement of one anolis sequence gave contradictory results. In the NJ tree the sequence is basal to the mammalian eutherian *Pgc1* (bootstrap 623), while in the ML tree the same sequence it comes basal to the *Pgb* clade (619 bootstrap). Based on the ML tree this sequence could represent a new gene lineage, which emerged in the ancestor of amniotes but was lost subsequently in birds, and mammals, with the reciprocal loss of *Pgc1* in reptiles. Four genes from the western clawed frog form an independent group which is basal to *Pgb* and *Pgc1* clades in both analyses, thus indicating that the *Pgb*/*Pgc1* duplication postdates amphibian divergence (Fig. 2). Both trees also display a third gene lineage found exclusively in birds (one gene), reptiles (one gene), amphibians (one gene) and marsupials (three genes), but not in eutherian mammals (Fig. 2A and Fig. 2B). The amphibian and bird case is particularly relevant, since these sequences were reported as *Pgc1* orthologues [10], [11]. However, our analysis clearly indicates that these gene sequences belong to a distinct gene lineage. The fourth anolis *Pgc-like* sequence which has a frameshift mutation in the eight exon, robustly groups with the reptile *Pgb* sequence (Figure S1), indicative of lineage specific duplication followed by loss. Finally, teleosts outgroup the full tetrapod gene collection. Based on the phylogenetic analysis, we introduce here a new gene nomenclature for *Pgc* genes in tetrapods which takes into account the evolutionary relationships between the various genes (Table 1 and Fig. 2). Thus, we maintain the designation for *Pgb* but modify the previous *Pgc* to *Pgc1*. The basal amphibian clade we name *Pgbc* (with an *1* to *4* nomenclature to designate each independent gene), and the new gene lineage emerging from the phylogeny is designated *Pgc2*. Teleost *Pgc* genes are named *Pgc*. In summary, our search identified at least four evolutionary independent gene lineages in tetrapods, *Pgb*, *Pgcb*, *Pgc1* and *Pgc2*. Independent gene expansions are observed at specific lineages in the amphibian and marsupial clades. Taking into account the duplication patterns emerging from the phylogenetic analysis, we anticipate also that various independent events of gene loss have taken place. That is the case of *Pgb* in some mammalian species (e.g. human), *Pgc2* in eutherian mammals, and *Pgc1* in birds.

### Tetrapod *Pgc* genes reside in a gene cluster

**Figure 3:**
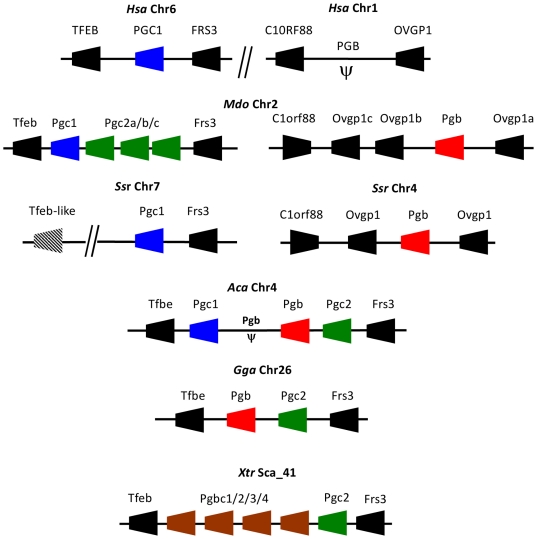
Synteny map of *Pgc loci* in tetrapods. Dashed gene box represents a TFEB partial ORF. Arrow head indicates gene orientation and *ψ* indicates pseudogene.

We next examined the genomic location of *Pgc* genes in tetrapods (Fig. 3), since it can provide powerful insights with respect to gene origin and loss. We find that *Pgc loci* are extremely well conserved between the various species, with two distinct settings. In basal tetrapods such as amphibians, chicken and anolis, we find the full *Pgc* gene portfolio mapping to a single location; while in mammals, *Pgc1* and *Pgb* genes reside at two distinct genomic locations (Fig. 3). We conclude that the expansion of the *Pgc* gene lineage in the ancestor of tetrapods occurred through tandem gene duplications. We further find that the *Pgb* translocation to a separate genomic *locus* is a more recent event which took place in the ancestor of mammals, in contrast to previous suggestions [6], since *Pgb* maps to the same *locus* in both the opossum and pig (similar to the *Pgb* pseudogene in humans).

**Figure 4:**
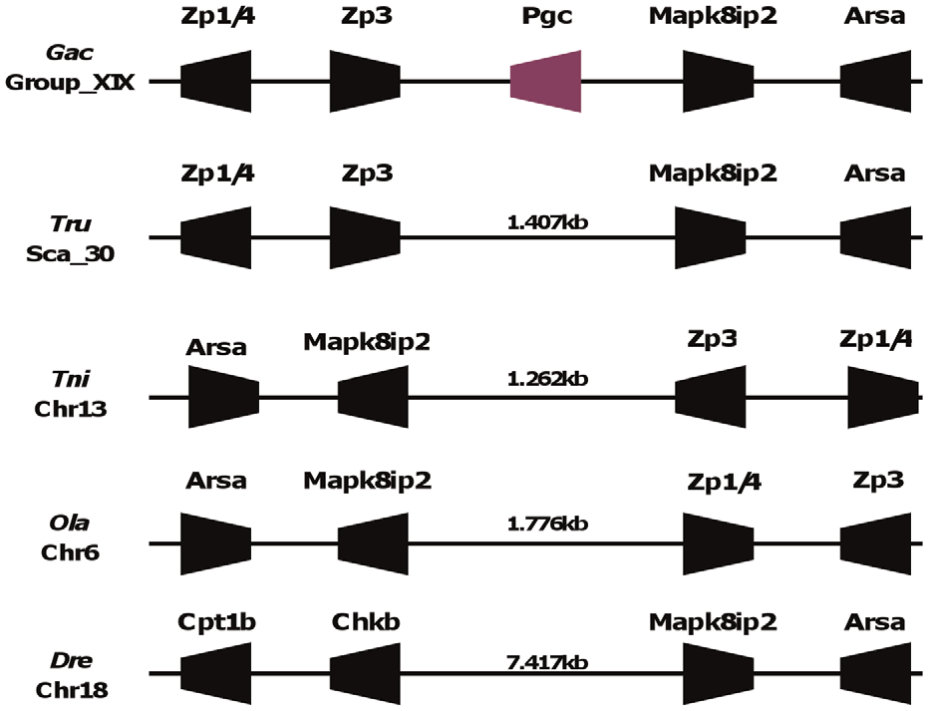
*Pgc loci* are conserved in teleost species, and indicate gene loss in some lineages. Gac – *G. aculeatus*, Tru- *T. rubripes*, Tni- *T. nigroviridis*, Ola- *O. latipes* and Dre- *D. rerio*. Numbers are distances between genes in Kb.

In contrast to tetrapods a single *Pgc* gene sequence has been described in various teleost species [9], a conclusion we now extent to stickleback. In this species, we find a single *Pgc* gene localizing to Group XIX (Fig. 4). A close inspection of the gene families flanking *GacPgc* shows no evidence of syntenic conservation in comparison to the tetrapod *Pgc locus*. We find for example that the stickleback orthologues of *Frs3* and *Tfeb* which outflank the *Pgc* gene cluster in tetrapods localize to Sca_27 in stickleback (not shown). Thus, *Pgc* has been apparently translocated from its original position in either tetrapods or teleosts and is of no evolutionary meaning. Mapping information from cartilaginous fish and pre-3R teleost species may provide insightful information on this issue. Except for the stickleback, we found no *Pgc-like* sequence in other teleost species with full genome sequences. To confirm the loss of *Pgc* sequences we analysed the composition of the *GacPgc locus* in zebrafish, medaka, pufferfish, and green pufferfish (Fig. 4). This approach confirms that neither of these species has a *Pgc* sequence in the genome (nor evidence for pseudogenization), despite the conservation of *locus* composition and organization.

## Discussion

**Figure 5:**
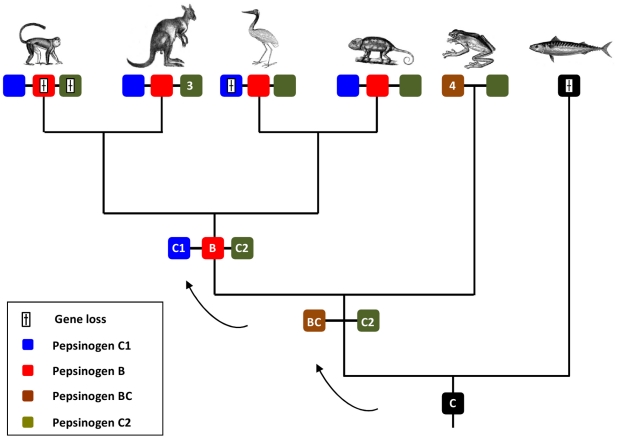
Proposed evolutionary history and duplication timings of the *Pgc* gene family in vertebrates. Numbers inside each box denotes gene numbers.

Here we analyse the evolutionary history of a gene family involved in the vertebrate gastric function, the *Pgc*, to find that extensive gene duplication and loss occurred in vertebrate classes. Our research begun by inquiring a long held premise that *Pgc* is a single copy gene family in vertebrate species [3], [9], [10], [11]. By taking an exhaustive search into various vertebrate genomes, we demonstrate that significant discrepancy in *Pgc* complements exists between species. For example, we find no *Pgc-like* gene in some teleost species (e.g. medaka), while up to five genes are found in the opossum and the western clawed frog. We next undertook a combination of phylogenetics and chromosomal gene location (and their neighbouring gene families) to reconstruct gene duplication timings and processes, relative to the divergence of major vertebrate classes. Our analysis supports an evolutionary scenario where tandem gene duplication and gene loss have dynamically taken place in the tetrapod lineage (Fig. 5). Consequently, we introduce a new gene nomenclature that incorporates the phylogenetic findings. Before the diversification of tetrapods, a gene duplication gave origin to two tandem paralogues, *Pgbc* and *Pgc2*. Preliminary data from the genome sequence of the coelacanth (*Latimeria chalumnae*) suggests that the duplication postdates the divergence of this basal Sarcopterygii lineage. *Pgc2* was maintained in most tetrapod species, but not in placental mammals. Episodes of lineage specific expansion were also observed in the opossum. As for the *Pgbc* gene, it expanded independently in the western clawed frog to held four gene copies (Fig. 5). Following the separation of amphibians but before amniote divergence, the *Pgbc* gene tandem duplicated to originate *Pgc1* and *Pgb* (Fig. 4), the latter being translocated from the *Pgc locus* in mammals. *Pgc1* was retained in most species, but not in the chicken and turkey, while *Pgb* experienced events of loss in some mammalian species, namely humans (Fig. 5). One anolis sequence (*Pgc1*) is inconsistently placed with both phylogenetic methods. In the NJ tree, it groups with the opossum *Pgc1* and basal to all other mammalian *Pgc1* genes. However, in the ML tree the same sequence is basal to the *Pgb* clade. If we consider the ML tree pattern correct, then this new gene represents a new lineage which emerged in the ancestor of amniotes but was lost subsequently in birds (1 event), and mammals (second event), plus the loss of *Pgc1* in the reptile. In contrast, the NJ tree requires less duplication and loss events. Thus, we consider more parsimonious to conclude that the anolis sequence is a true *Pgc1* gene. The position of the sequence in the *Pgc* gene cluster is also in agreement with this interpretation. Although this is only indicative evidence, this gene maps on the side of *Tfeb*, just as is observed in other species.

Surprisingly, *Pgc* has not been retained in every examined fish species (Fig. 4, Fig. 5). We find that some teleosts have no representative of *Pgc* in their genome, though other pepsinogen families can be found [33]. Documenting patterns of gene loss is of extreme relevance, particularly for the understanding of phenotypic evolution [34]. Furthermore, gene loss has been correlated with the evolution of functional changes in surviving gene family members [35]. Currently, it is unclear whether the loss of *Pgc* genes in some teleosts and that of other *Pgc-like* lineages in tetrapods (e.g. *Pgb*) affected the evolution of additional pepsinogen gene family members (e.g. *Pga*), as well as, the gastric function.

Gene duplication is major source of morphological and functional innovation. The retention of the descendent gene copies can lead to the partitioning of ancestral functions or alternatively to the emergence of novel roles [36], [37]. The finding of different *Pgc* gene lineages (and complements) in vertebrate classes suggests that functional divergence took place between isoforms. It has been argued that pepsinogen gene expansion, namely in *Pga*, enabled the appearance of proteins with different specificities, being advantageous for effective gastric digestion [7]. Experimental assays with PGC1 and PGB have hinted at distinct functional profiles. For example, porcine PGB hydrolytic activity towards haemoglobin is residual, when compared to human PGC1 [3]. Also, teleost *Pgc* shows minor specific activity towards haemoglobin as well [9]. The analysis of the dog PGB peptide cleavage capacity demonstrated preference for Phe–X bonds while PGC1 cleaves Tyr-X bonds [3], [24]. In PGB, Tyr13 and Phe221 were shown to be crucially involved in substrate specificity; molecular modeling of pepsin B demonstrated that these residues lead to distinct network of hydrogen bonds and consequently accommodate different substrates in the binding cleft [25]. For other *Pgc* isoforms described here no experimental data is yet available. However, despite the high conservation degree at proposed critical enzymatic residues some sequence differences are discernible (Fig. 1). Several non-conserved residues located in the S1 and S1′subsites and their vicinities suggest subtle structural changes, namely in the enzyme structure, exposure of the active aspartic residues and in the general architecture of the binding cleft (Figure S2 and details therein). Thus, we propose the *Pgc* gene expansion was accompanied by the acquisition of novel substrate specificities.

The expansion of *Pgc* gene family finds parallel in other pepsinogen gene families, namely *Pga* [3], [7]. In hominoids, two separate *Pga* lineages (and isoform numbers) have been described, *Pga1* and *Pga2* [7]. Interestingly, *Pga1* and *Pga2* have rather distinct PI values suggesting activation at different pHs, analogous to our findings in *Pgc* (Table 1). Also, it has been argued that the expansion of *Pga* might be advantageous for gastric digestion [7], with the multiplicity of *Pga* genes apparently linked to food habits [7]. Two fold reasons support this hypothesis: a higher number of *Pga* genes contributes to a higher level of pepsin in the stomach, and distinct isoforms (A1 and A2) have evolved distinct proteolytic specificities [7]. Coincidently, the majority of the gene expansion events observed in the *Pgc* gene family notably coincides with the invasion of terrestrial habitats. In effect, the extensive increase of *Pgc* gene lineages and independent expansions is uniquely observed in tetrapods, at two distinct moments, before the divergence of amphibians and amniotes respectively. Thus, we propose that the access to new dietary protein sources acted as the driving force for *Pgc* retention and functional diversification after gene duplication. Conversely, the targeted loss of some isoforms, such as the *Pgb* in some mammalian species or *Pgc1* in birds, once more resulted from changes in protein sources which rendered the retention of some *Pgc* isoforms less important.

### Conclusions

The data presented here significantly modifies our knowledge about the overall evolutionary history of the *Pgc* gene family considered so far. We show that *Pgc* has undergone episodes of expansion, loss and retention. We conclude that tandem duplications have modelled the history of *Pgc* genes, probably underscoring different enzymatic requirements and specificities towards protein dietary sources. Future experimental assays should take into account the evolutionary history and diversity of *Pgc* genes in vertebrates.

## Supporting Information

Figure S1 ***Anolis carolinensis Pgb-like*** **pseudogene.** Grey and white shading indicate exon boundaries. In panel **A** *Anolis carolinensis* pseudogene PGB gene cDNA (Gene ID: 100567523). Highlighted in red we find the insertion of a guanine producing a premature stop codon downstream also in red. In Panel **B** Translation *Anolis carolinensis* pseudogene PGB gene cDNA, asterisk indicates stop codon. A frameshift mutation upstream results in a premature stop codon observed in exon 8. In Panel **C**, we provide a schematic representation of the *Anolis carolinensis* pseudo gene organization. Below, in detail a partial sequence alignment of exon 8 from *Anolis carolinensis* PGC1 (Ac-PGC1) *Anolis carolinensis pseudogene* (Ac-PGBΨ) and *Homo sapiens* PGC1 (Hs-PGC1). Highlighted in red frame shift mutation caused by the insertion of an guanine leading to a premature stop codon downstream also highlighted in red. Panel **D** NJ tree showing that the *AcPgb* pseudogene robustly groups with the *Pgb* orthologue.
(PPT)

Figure S2 **Structural analysis of the PGC sequences suggests distinct substrate specificities.** Hs - *Homo sapiens*; Md - *Monodelphis domestica*; Ss - *Sus scrofa*; Ac - *Anolis carolinensis*; Xt - *Xenopus tropicalis*; Ga - *Gasterosteus aculeatus*, To - *Thunnus orientalis*. All pepsinogen 3D theoretical models present a bilobal structure with the substrate binding cleft located in the middle of the two lobes (Panel A small image, 1- N-terminal; 2-substrate binding cleft and 3- C-terminal). Red corresponds to the location of the S1 subsite residues. Dark green corresponds to the S1′subite and lime green corresponds to the Asp32 and Asp217 residues. Models show a highly similar 3D structure within each PGC group (e.g. PGC1) in contrast, when comparing between groups (e.g. PGC1 and PGC2) it is possible to detect subtle differences in the enzyme structure, such as location of the S1 and S1′subsites, exposure of the active aspartic residues and in the general architecture of the binding cleft. In Panel (A) the hsPGC corresponds to the 1HTR crystal structure available at Protein Database (PDB), and which is highly similar to other PGC models presented, at position 7 we observe a methionine that impacts the cleft structure and is located near Asp32. In panel (B) three models of PGB are presented, at the equivalent position these models present an Isoleucine or and Phenylalanine which are bulky hydrophobic residues that may contribute to the narrowing of the cleft. In panel (C) PGBC models also present a subtle enlargement of the cleft possibly due to the distinct orientation of the methionine residue at position 7. In panel (D) PGC2 models show that the catalytic aspartic residues are more exposed in comparison to other PGC proteins and these models also present a larger cleft possibly due to an alternative Leucine residue at position 7. In panel (E) we observe that fish PGC models present a small N-terminal region in comparison with the other models. It is possible to observe that the subite S1 is located further from the active site in comparison with the other models, finally due to a deletion in the sequence fish PGC present an non hydrophobic asparagine residue at position 7 opposing the hydrophobic residues encountered at this location, this is a comparatively small residue possibly leading to an enlargement of substrate binding cleft in this region.
(TIF)
