# Supplementary material for: The Evolution of Pepsinogen C Genes in Vertebrates: Duplication, Loss and Functional Diversification
Source: PLoS One. 2012 Mar 9;7(3):e32852. doi: 10.1371/journal.pone.0032852 (PMC3298455; doi:10.1371/journal.pone.0032852)
Supplement: Figure S1 — Anolis carolinensis Pgb-like pseudogene. Grey and white shading indicate exon boundaries. In panel A Anolis carolinensis pseudogene PGB gene cDNA (Gene ID: 100567523). Highlighted in red we find the insertion of a guanine producing a premature stop codon downstream also in red. In Panel B Translation Anolis carolinensis pseudogene PGB gene cDNA, asterisk indicates stop codon. A frameshift mutation upstream results in a premature stop codon observed in exon 8. In Panel C, we provide a schematic representation of the Anolis carolinensis pseudo gene organization. Below, in detail a partial sequence alignment of exon 8 from Anolis carolinensis PGC1 (Ac-PGC1) Anolis carolinensis pseudogene (Ac-PGBΨ) and Homo sapiens PGC1 (Hs-PGC1). Highlighted in red frame shift mutation caused by the insertion of an guanine leading to a premature stop codon downstream also highlighted in red. Panel D NJ tree showing that the AcPgb pseudogene robustly groups with the Pgb orthologue. (PPT) [file pone.0032852.s001.ppt]

## Slide 1
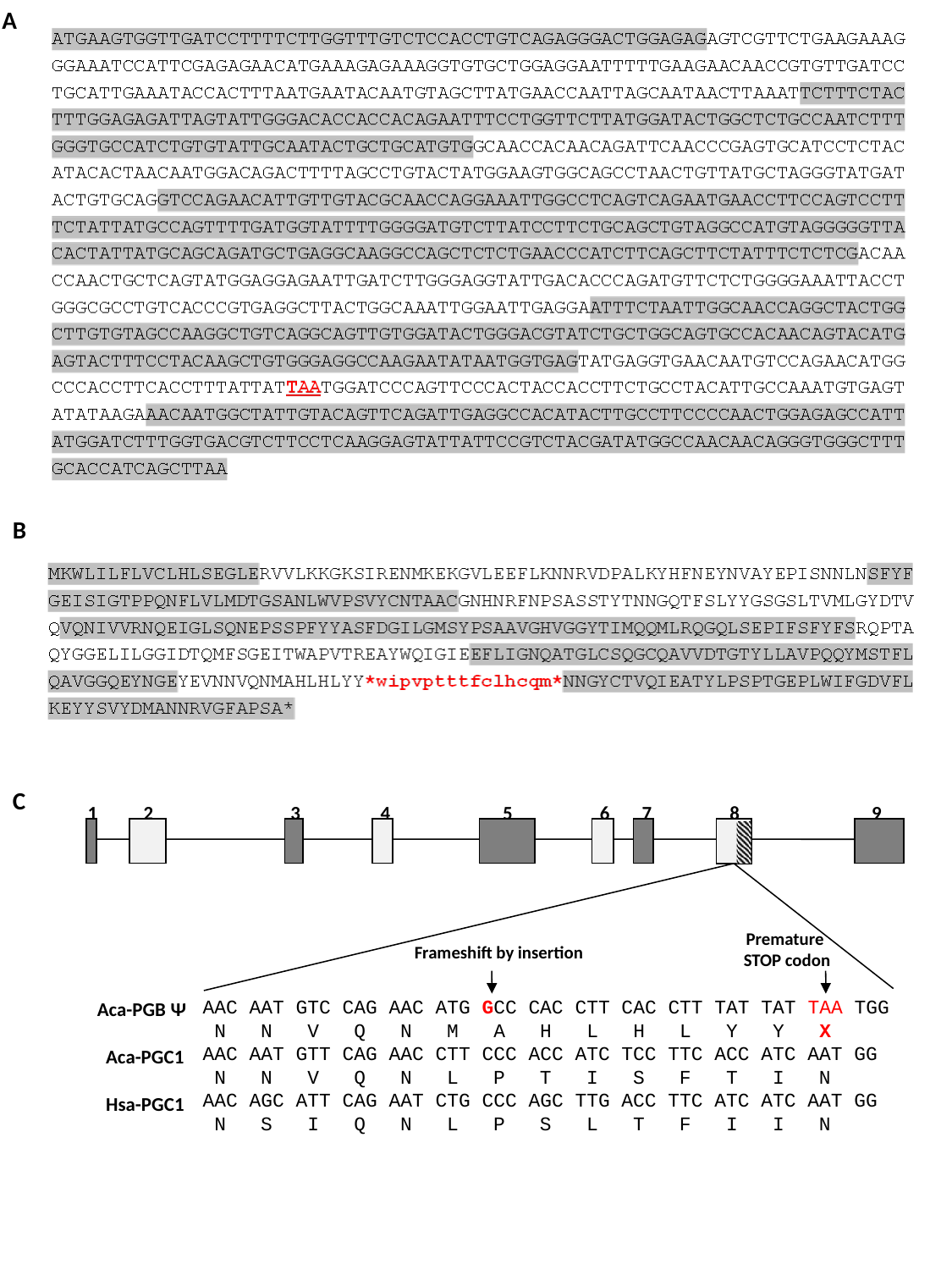

A
B
C
1
2
3
4
5
6
7
8
9
AAC AAT GTC CAG AAC ATG GCC CAC CTT CAC CTT TAT TAT TAA TGG
 N N V Q N M A H L H L Y Y X
AAC AAT GTT CAG AAC CTT CCC ACC ATC TCC TTC ACC ATC AAT GG
 N N V Q N L P T I S F T I N
AAC AGC ATT CAG AAT CTG CCC AGC TTG ACC TTC ATC ATC AAT GG
 N S I Q N L P S L T F I I N
Aca-PGB Ѱ
Aca-PGC1
Hsa-PGC1
Premature
STOP codon
Frameshift by insertion

## Slide 2
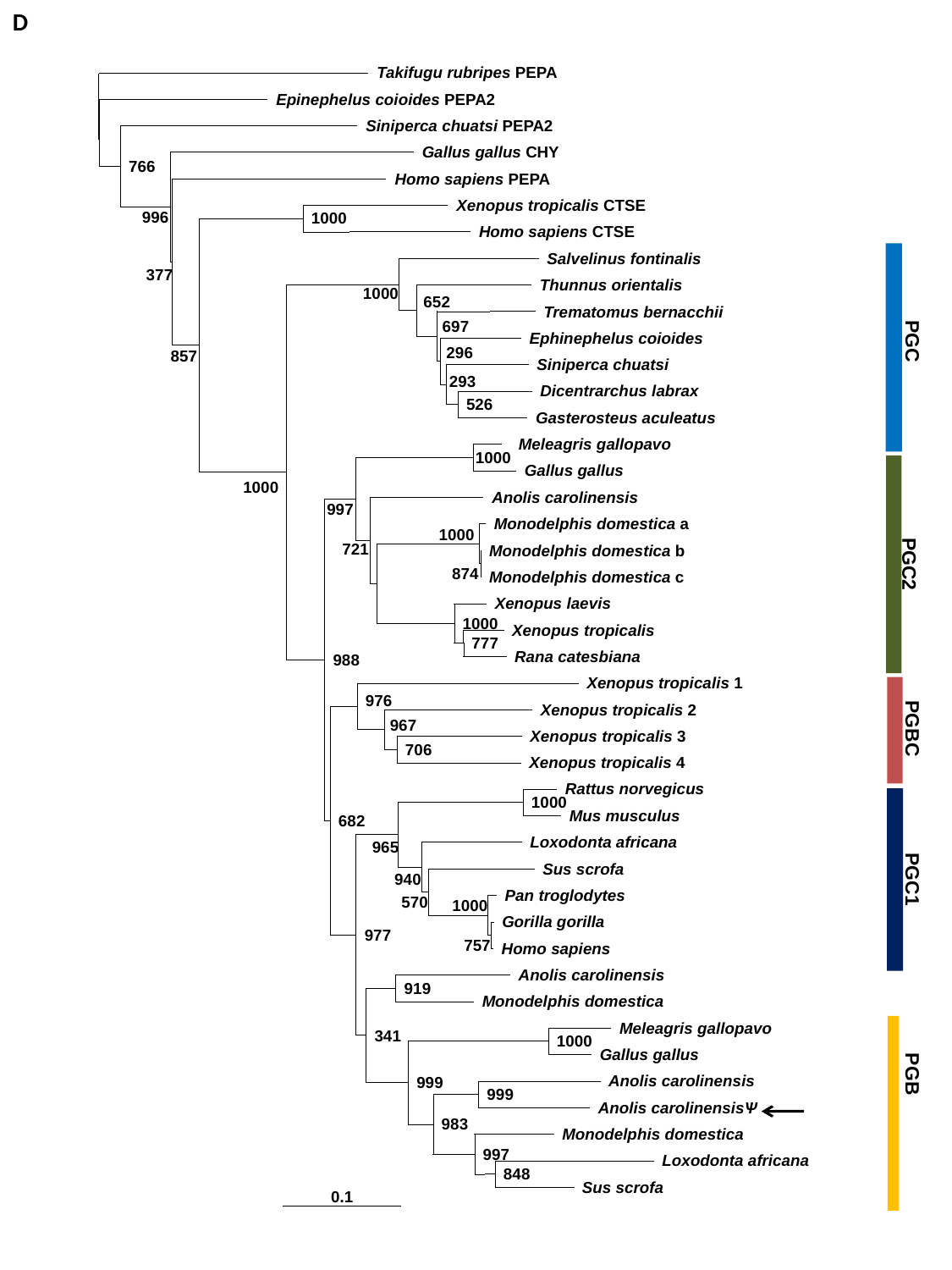

D
Takifugu rubripes PEPA
Epinephelus coioides PEPA2
Siniperca chuatsi PEPA2
Gallus gallus CHY
766
Homo sapiens PEPA
Xenopus tropicalis CTSE
996
1000
Homo sapiens CTSE
Salvelinus fontinalis
377
Thunnus orientalis
1000
652
Trematomus bernacchii
697
Ephinephelus coioides
296
857
Siniperca chuatsi
293
Dicentrarchus labrax
526
Gasterosteus aculeatus
Meleagris gallopavo
1000
Gallus gallus
1000
Anolis carolinensis
997
Monodelphis domestica a
1000
721
Monodelphis domestica b
874
Monodelphis domestica c
Xenopus laevis
1000
Xenopus tropicalis
777
Rana catesbiana
988
Xenopus tropicalis 1
976
Xenopus tropicalis 2
967
Xenopus tropicalis 3
706
Xenopus tropicalis 4
Rattus norvegicus
1000
Mus musculus
682
Loxodonta africana
965
Sus scrofa
940
Pan troglodytes
570
1000
Gorilla gorilla
977
757
Homo sapiens
Anolis carolinensis
919
Monodelphis domestica
Meleagris gallopavo
341
1000
Gallus gallus
Anolis carolinensis
999
999
Anolis carolinensisѰ
983
Monodelphis domestica
997
Loxodonta africana
848
Sus scrofa
0.1
PGC
PGC2
PGBC
PGC1
PGB
