# Supplementary figures and images for: The Evolution of Pepsinogen C Genes in Vertebrates: Duplication, Loss and Functional Diversification
Source: PLoS One. 2012 Mar 9;7(3):e32852. doi: 10.1371/journal.pone.0032852 (PMC3298455; doi:10.1371/journal.pone.0032852)

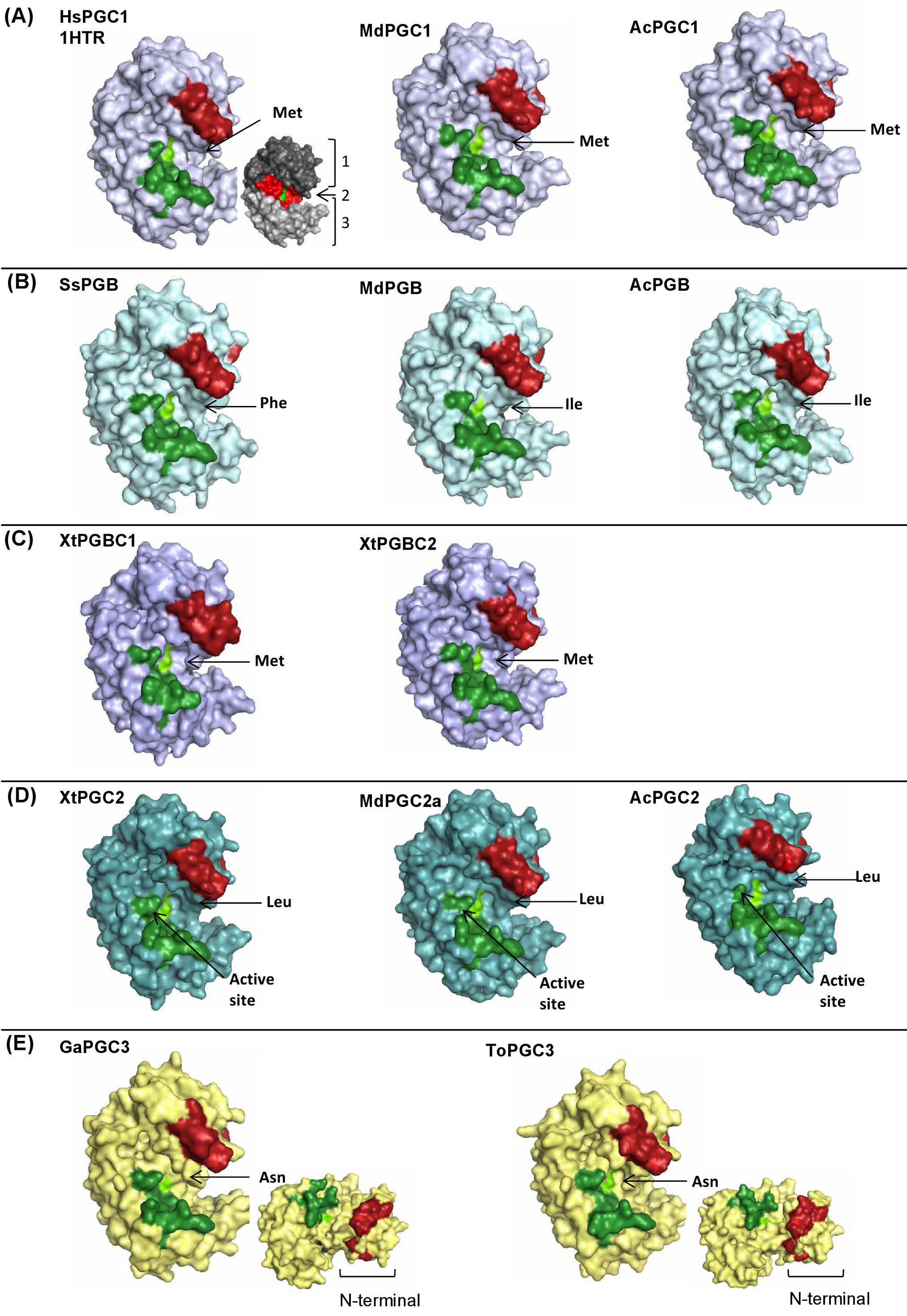

Supplement: Figure S2 — Structural analysis of the PGC sequences suggests distinct substrate specificities. Hs - Homo sapiens; Md - Monodelphis domestica; Ss - Sus scrofa; Ac - Anolis carolinensis; Xt - Xenopus tropicalis; Ga - Gasterosteus aculeatus, To - Thunnus orientalis. All pepsinogen 3D theoretical models present a bilobal structure with the substrate binding cleft located in the middle of the two lobes (Panel A small image, 1- N-terminal; 2-substrate binding cleft and 3- C-terminal). Red corresponds to the location of the S1 subsite residues. Dark green corresponds to the S1′subite and lime green corresponds to the Asp32 and Asp217 residues. Models show a highly similar 3D structure within each PGC group (e.g. PGC1) in contrast, when comparing between groups (e.g. PGC1 and PGC2) it is possible to detect subtle differences in the enzyme structure, such as location of the S1 and S1′subsites, exposure of the active aspartic residues and in the general architecture of the binding cleft. In Panel (A) the hsPGC corresponds to the 1HTR crystal structure available at Protein Database (PDB), and which is highly similar to other PGC models presented, at position 7 we observe a methionine that impacts the cleft structure and is located near Asp32. In panel (B) three models of PGB are presented, at the equivalent position these models present an Isoleucine or and Phenylalanine which are bulky hydrophobic residues that may contribute to the narrowing of the cleft. In panel (C) PGBC models also present a subtle enlargement of the cleft possibly due to the distinct orientation of the methionine residue at position 7. In panel (D) PGC2 models show that the catalytic aspartic residues are more exposed in comparison to other PGC proteins and these models also present a larger cleft possibly due to an alternative Leucine residue at position 7. In panel (E) we observe that fish PGC models present a small N-terminal region in comparison with the other models. It is possible to observe that the subite S [file pone.0032852.s002.tif]
